# Severe malaria in Gabon: epidemiological, clinical and laboratory features in Amissa Bongo Hospital of Franceville

**DOI:** 10.1186/s12936-023-04512-7

**Published:** 2023-03-09

**Authors:** Roméo Karl Imboumy-Limoukou, Judicael Boris Lendongo-Wombo, Andhra Fecilia Nguimbyangue-Apangome, Jean-Claude Biteghe Bi Essone, Franck Mounioko, Lydie Sandrine Oyegue-Libagui, Brice Edgar Ngoungou, Jean-Bernard Lekana-Douki

**Affiliations:** 1 Unité Ecologie Evolution et Résistances Parasitaires (UNEEREP), Centre International de Recherches Médicales de Francevillev, BP 769, Franceville, Gabon; 2 Ecole Doctorale Régionale d’Afrique Centrale en Infectiologie Tropicale (ECODRAC), Université Des Sciences Et Techniques de Masuku, BP 876, Franceville, Gabon; 3 Département de Parasitologie-Mycologie, Université Des Sciences de La Santé, BP 18231, Libreville, Gabon; 4 Département d’épidémiologie, Biostatistiques Et Informatique Médicale, Université Des Sciences de La Santé (USS), BP 18231, Libreville, Gabon; 5 Département de Biologie, Université Des Sciences Et Techniques de Masuku (USTM), BP 901, Franceville, Gabon

**Keywords:** Severe malaria, Falciparum, Emergency ward, Gabon

## Abstract

**Background:**

Malaria is the most deadly parasitic disease and continues to claim more than a half million of deaths across the world each year, mainly those of under-fives children in sub-Saharan Africa. The aim of this study was to determine the epidemiological, clinical and laboratory features of patients with severe malaria at the Centre Hospitalier Régional Amissa Bongo (CHRAB), a referral hospital in Franceville.

**Methods:**

It was an observational descriptive study conducted at CHRAB over 10 months. All admitted patients at the emergency ward of all ages presenting with positive test to falciparum malaria diagnosed by microscopy and rapid test with clinical signs of severe illness describe by World Health Organization were enrolled.

**Results:**

During this study, 1065 patients were tested positive for malaria, of them 220 had severe malaria. Three quarters (75.0%) were less than 5 years of age. The mean time to consultation was 3.5 ± 1 days. The most frequent signs of severity on admission were dominated by neurological disorders 92.27% (prostration 58.6% and convulsion 24.1%), followed by severe anemia 72.7%, hyperlactatemia 54.6%, jaundice 25% and respiratory distress 21.82%.The other forms such as hypoglycemia, haemoglobinuria, renal failure were found in low proportions < 10%. Twenty-one patients died, coma (aOR = 15.54, CI 5.43–44.41, p < 0.01), hypoglycemia (aOR = 15.37, CI 2.17–65.3, p < 0.01), respiratory distress (aOR = 3.85, CI 1.53–9.73, p = 0.004) and abnormal bleeding (aOR = 16.42, CI 3.57–104.73, p = 0.003) were identified as independent predictors of a fatal outcome. Anemia was associated with decreased mortality.

**Conclusion:**

Severe malaria remains a public health problem affecting mostly children under 5 years. Classification of malaria helps identify the most severely ill patients and aids early and appropriate management of the severe malaria cases.

## Background

In 2020, malaria remains a devastating health problem and the deadliest parasitic disease for humans. Nearly 241 million cases of malaria occurred worldwide in 2020, most of which were in the WHO African Region. The same year, 627,000 malaria-related deaths were recorded [1]. Children under 5 years old are the most vulnerable group affected by malaria; in 2019, children accounted for 67% of all malaria deaths worldwide. *Plasmodium* infections result in a spectrum of clinical effects, including asymptomatic parasitemia, uncomplicated malaria, severe malaria, and death [2]. Of the five different species of *Plasmodium* which can infect humans, *Plasmodium falciparum* is the most pathogenic species and accounts for the majority of severe and fatal malaria in Africa and particularly in Gabon [3, 4], principally due to endothelial cytoadherence causing sequestration of mature-staged infected red blood cells in vital organs [5, 6]. In endemic areas, young children and pregnant women are the most vulnerable to severe malaria while older children and adults develop partial immunity after repeated infections and thus have lower risk of severe disease [7].

Severe falciparum malaria definition (SFM) has evolved these last two decades. In 1990, the World Health Organization (WHO) established criteria for the diagnosis of severe malaria [8]. In 2000, the WHO revised these criteria to include other clinical manifestations and laboratory values that portend a poor prognosis based on clinical experience in semi‑immune patients. The last update of SFM definition established that SFM is defined by the detection of *P. falciparum* by microscopy or a rapid diagnostic test and at least one criterion for severe disease (impaired consciousness, respiratory distress, multiple convulsions, prostration, shock, pulmonary oedema, abnormal bleeding, jaundice, severe anemia, hypoglycaemia, acidosis, hyperlactataemia, renal impairment, or hyperparasitaemia) [8].

Malaria transmission in Gabon is stable and perennial [3]. There is limited data describing severe falciparum malaria in Gabon and no study has yet been done in Franceville. Furthermore studies conducted on the topic did not take into account the last updates of SFM definition. This is the first study in Gabon that takes account of the last updates of WHO severe malaria criterions. The new WHO updates of severe malaria can help to better characterize malaria signs and symptoms, help clinicians avoid delays in diagnosis, identify patients who are more likely to die and thus improve their management.

## Methods

### Study site

This study was conducted at the emergency ward of Centre Hospitalier Régional Amissa Bongo (CHRAB). The CHRAB is located in the town of Franceville and it is the most important public healthcare facility in the Haut-Ogooué province. Franceville is an urban region of south-east Gabon (1° 37′ 15″ S, 13° 34′ 58″ E), the capital city of Haut-Ogooué, it is the third main town of Gabon in terms of population (pop. 110, 568 hab) [4]. Malaria prevalence is around 20% [5].

### Study period

The study was carried out from June 2019 to April 2020 (03 June 2019–09 April 2020).

### Patients and procedures

All febrile (or those with history of fever in the last 48 h) participants admitted at the emergency ward and pediatric emergency ward of all ages were referred to the study team and seen on admission by a clinical staff of the study. Summary data were recorded on a pro-forma sheet. Only patients presenting with positive falciparum malaria test with clinical signs of severe illness (as described above, according to the WHO criteria [8]) were enrolled. These patients were examined by a physician and their data captured on a standardized form. The study was authorized by the Director of the hospital and approved by the Gabonese National Ethics Committee (PROTN°23/2019/PR/SG/CNER). Only children whose parents gave informed written consent were enrolled in the study. The rainfall dataset for this study was obtained from the Agence pour la sécurité de la navigation aérienne en Afrique et à Madagascar (ASECNA).Venous blood was collected for all enrolled patients for the following medical exam.

### Laboratory procedures

Laboratory analysis were performed at the CHRAB’s laboratory and the Centre International de Recherches Médicales de Franceville (CIRMF). Malaria was confirmed by thick blood film under the light microscope. The investigations were performed within 30 min of blood collection at the CHRAB’s laboratory. Thick blood films were stained with 3% Giemsa and parasite load was determined using the Lambarene method [6]. All thick blood were read by two independent qualified microscopists and quality control was done in 10% of slides by a third reader. The Optimal-IT^®^ rapid diagnostic test detecting *P. falciparum* was also used according to the manufacturers’ instructions [7]. A previous study led in Gabon evaluated this RDT and showed that it is a good tool for malaria diagnosis [9]. Measurement of haemoglobin concentration and white cell count were carried out using an automate ABX Micros^®^ (Horiba, Japan) from EDTA blood samples. Capillary blood glucose was measured with an ACCU-CHEK^®^ Active blood glucose meter (Roche, South Africa). Biochemical analyses were carried out by an ABX PENTRA device (Horiba, Japan) on plasma samples. Macroscopic haemoglobinuria was measured using SD UroColor^®^ Urine Test Strip (SD, South Korea).

### Chest radiography

The chest X-ray was performed when clinical examination is suggestive of lung damage at the CHRAB radiography department.

### Case definitions

Severe malaria was defined by the detection of *P. falciparum* by microscopy or a rapid diagnostic test and at least one WHO criterion for severe disease (see above). Fever was defined as axillary temperature  ≥ 37.5 °C or reported history of fever in the past 48 h.

### Statistical analysis

Data of patients were recorded in Excel 2013 spreadsheets. Statistical analysis was performed using the Epi-Info 6 and R version 4.0.5 (2021-03-31) software. Simple proportion were calculated for qualitative variables and quantitative variables by mean, standard deviation (SD), median with inter-quartile range (IQR). The qualitative variables were compared using the Chi-square test or Fisher’s exact test for numbers below 5. Binary logistic regression model was used to compute the adjusted odds ratios (aOR) of independent risk factors. The confidence interval was set at 95% (95% CI). Statistical significance was set at α = 5%.

## Results

### Demographic and clinical data of patients included

**Fig. 1:**
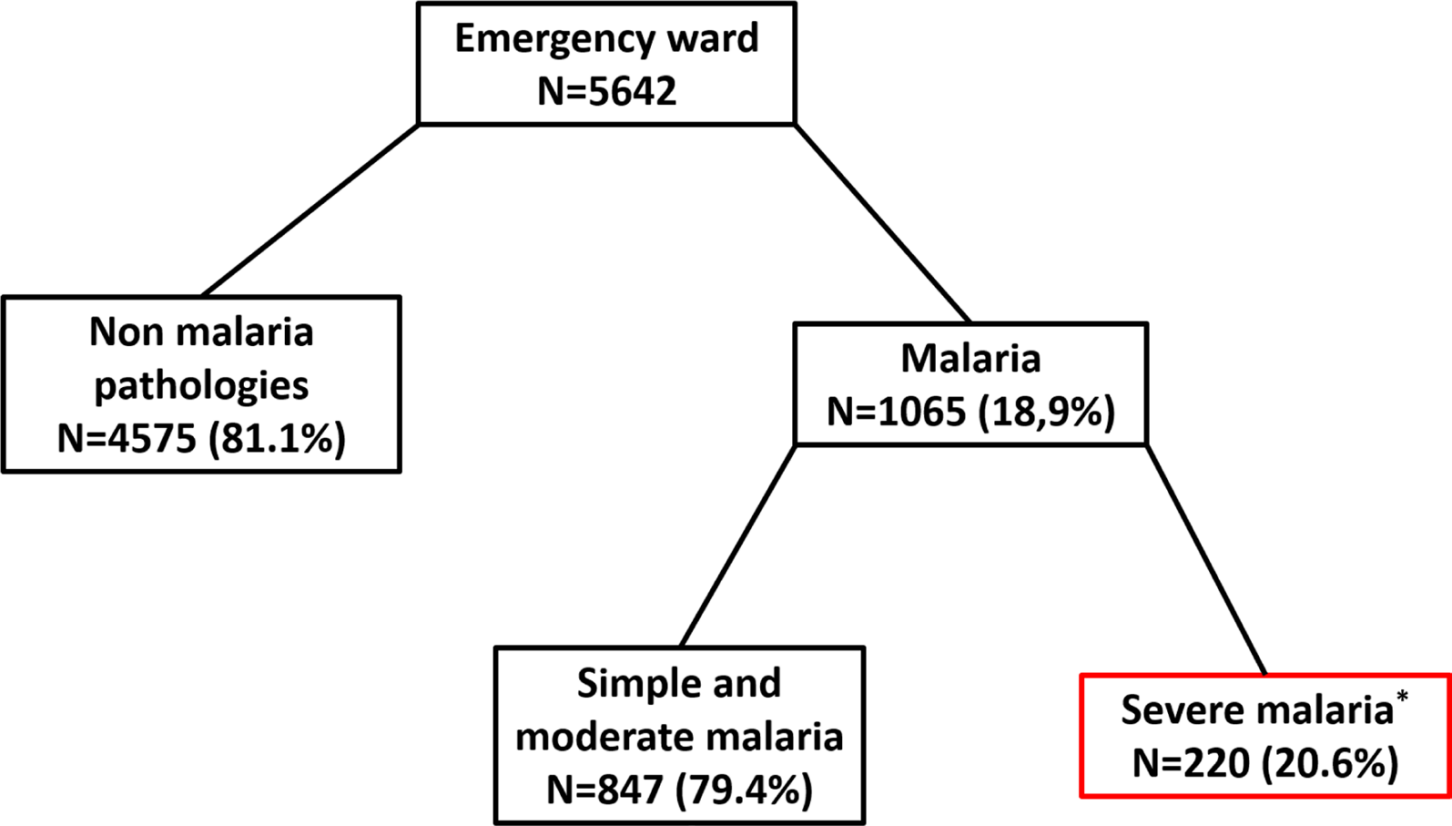
Flowchart of the screening of severe malaria cases in the CHRAB’s emergency ward. *According to the WHO criterions

From June 2019 to March 2020, a total of 5642 patients were admitted to the CHRAB emergency department. One thousand sixty-five (1065) of them were tested positive for malaria and the 4575 other patients were admitted for another pathology. Of 1065 patients with malaria, 847 (79.4%) had simple or moderate malaria and 220 (20.6%) had severe malaria (Fig. 1).

Demographic data and individual factor for patients are represented in Table 1. Patients included in this study ranged in age from 3 to 1128 months (94 years) with a sex ratio (M/F) of 1.09. Three quarters (165/220, 75.0%) of the patients were less than 5 years of age.

**Table 1:** Demographic characteristics and individual factors of patients

General characteristics	
Number	220
Mean temperature (SD)	38.4 (0.97)
Parasitemia	
Mean	129583 (200917)
Median [IQR]	44800 [5500–156800]
Gender	
Male, n (%)	115 (52.27)
Female, n (%)	105 (47.73)
Age	
Mean age (SD)	55.2 (88.6)
Median [IQR]	36 [19.8–60.0]
Age groups stratified	
0–5, n (%)	165 (75)
5–10, n (%)	36 (16.40)
10–15, n (%)	13 (5.90)
> 15, n (%)	06 (2.70)
Education level of patients/parents/guardians	
Primaryschool	48 (21.82)
High school	169 (76.82)
University	3 (1.36)
Income-generating activities	
Yes, n (%)	151 (68.64)
No, n (%)	69 (31.36)
Residence	
Urban, n (%)	132 (60)
Rural, n (%)	88 (40)

### Seasonal distribution of severe malaria case

**Fig. 2:**
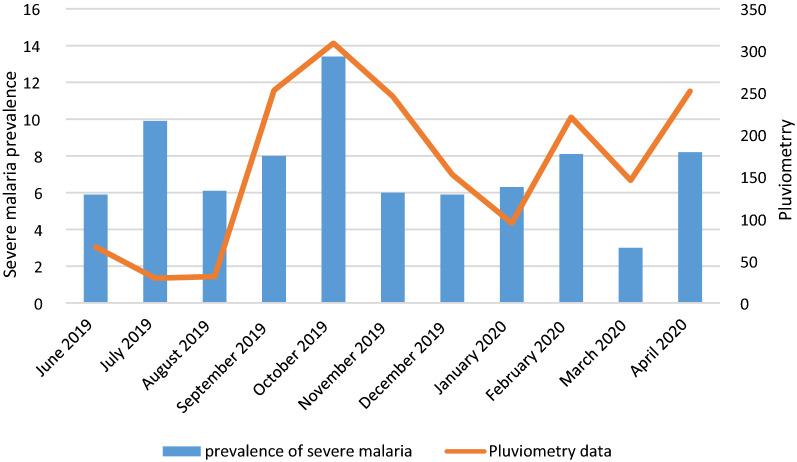
Distribution of the severe malaria cases according to the pluviometry

The monthly distribution of severe malaria cases was analyzed in relation to rainfall (Fig. 2). The prevalence of severe malaria cases differs by month (p = 0.007). The majority of severe malaria cases were observed during the months of September 2019 to November 2019 (short rainy season). Mars 2020 had the lowest proportion of severe malaria cases.

### Self-medication and patient consultation

The most commonly used antimalarial drug for self-medication was artemisinin-based combination therapy 171/220 (77.7%) as show in Table 2.

**Table 2:** Use of self-medication practices

Preventives measures	
Practice of self-medication	
Number	220
Yes, n (%)	171 (77.7)
No, n (%)	24 (10.9)
Don't know, n (%)	25 (11.4)
Anti-malarial drugs used in self-medication	
Number	171
ACT^*^, n (%)	99 (57.8)
Quinines, n (%)	33 (19.30)
Sulfadoxine-pyrimethamine, n (%)	1 (0.58)
Other (non-antimalarial drug)	38 (22.22)

^*^ACT artemisinin-based combination therapy

### Patient consultation time and death

No patient with severe malaria went to the hospital at the start of symptoms. The mean time to consultation was 3.5 ± 1 days (extremes 1–7 days). Patients with severe malaria usually had a delay in consultation of more than 48 h (53.64%). Time to consultation from symptoms onset was not associated with death (p = 0.13). A total of 21 (9.54%) patients died during study. Sixteen (76.19%) patients died less than 24 h after their hospitalization (Table 3).

**Table 3:** The consultation time in relation to severe falciparum malaria death

Delay of consultation	Number (%) (N = 220)	Died survived		*p*-value
		n = 21	n = 199	
24–48 h	102 (46.36)	13(12.74)	55 (87.15)	*0.13*
> 48 h	118 (53.64)	8 (6.78)	110 (93.22)	

### Clinical and biological description of severe malaria

All severe clinical forms of severe *P*. *falciparum* malaria are shown in Table 4. The neurological disorders accounted for 92.27% of cases (prostration (58.6%; n = 129), repeated convulsions (24.1%; n = 53) and coma (9.5%; n = 21)). Pulmonary oedema, macroscopic haemoglobinuria and abnormal bleeding were rarely observed. The laboratory features observed in the patients were dominated by severe anaemia (72.7%; 160/220), hyperlactataemia (54.6; 59/108) and hyperparasitaemia (5.45%; 12/220). The other laboratory features observed in the patients included hyperbilirubinaemia (9.3%; 8/86), rarely hypoglycaemia and hypercreatinaemia. Of 220 patients, 21 died, giving an overall case fatality rate of 9.5%. The full clinical and laboratory features are shown in Table 4.

**Table 4:** Clinical and biological features of severe falciparum malaria and relation to death of patient

Parameters	*n*	*%*	Died (n = 21)	Survived (n = 199)	*p*-value
*Clinical parameters*					
Prostration, n (%)	129	58.6	11 (8.53)	118 (91.47)	0.54
Convulsions, n (%)	53	24.1	8 (15.09)	45 (84.91)	0.11
Coma, n (%)	21	9.5	10 (47.62)	11 (52.38)	0.001
Respiratory distress, n (%)	48	21.82	10 (20.83)	38 (79.17)	0.01
Jaundice, n (%)	55	25	8 (14.55)	47 (85.45)	0.15
Pulmonary oedema, n (%)	2/48^**^	4.17	0 (0)	2 (100)	–
Macroscopic haemoglobinuria, n (%)	10	4.55	1 (10)	9 (90)	1
Circulatory collapse, n (%)	3	1.36	1 (33.33)	2 (66.67)	–
Anormal bleeding, n (%)	5	2.27	2 (40)	3 (60)	< 0.01
*Biological parameters*					
Severe anaemia, n (%)	160	72.7	11 (6.88)	149 (93.12)	0.028
Hyperparasitaemia, n (%)	12	5.45	2 (16.67)	10 (83.33)	0.31
Hypoglycaemia, n (%)	7	3.18	4 (57.14)	3 (42.86)	< 0.01
Hyperbilirubin, n (%)	8/86^*^	9.3			
Hyperlactataemia, n (%)	59/108^*^	54.6	6 (10.17)	53 (89.83)	1
Renal failure, n (%)	2/212^*^	0.94	2 (100)	0 (0)	–

^**^X-ray were performed only in patients with evocative signs

^*^Due to the availability of reagents, these exams were not performed in all patients

### Individual characteristics data of patients in relation to death

Table 5 presents the social characteristics of the patients in relation to death. The number of deaths related to severe malaria differed according to age group (*p* = 0.019). The highest number of deaths was observed in the age group of 0–5 years.

**Table 5:** Analyses of social characteristics according to death

	*n* (*N* = 220)	Died (n = 21)	Survived (n = 199)	*p*-value
Self-treatment, n (%)	171	15 (8.77)	136 (79.53)	0.51
Age groups stratified, n (%)				
0–5 years, n (%)	165	14 (8.48)	151 (91.52)	0.019
5–10 years, n (%)	36	2 (5.56)	34 (94.44)	–
10–15 years, n (%)	13	2 (15.38)	11 (84.62)	–
> 15 years, n (%)	6	3 (50)	3 (50)	–
Place of residence				
Urban, n (%)	132	14 (10.61)	118 (89.39)	0.51
Rural, n (%)	88	7 (7.95)	81 (92.05)	–
Gender				
Male, n (%)	115	13 (11.30)	102 (88.70)	0.35
Female, n (%)	105	8 (7.62)	97 (92.38)	–
Income-generating activities				
Yes, n (%)	69	5 (7.24)	64 (92.75)	0.43
No, n (%)	151	16 (10.60)	135 (89.40)	–

### Association of severe malaria cases with death

Table 6 shows a multiple logistic regression model identifying coma (aOR = 15.54, CI 5.43–44.41, p < 0.01), hypoglycaemia (aOR = 15.37, CI 0.96–0.99, p < 0.01), respiratory distress (aOR = 3.85, CI 1.53–9.73, p = 0.004) and abnormal bleeding (aOR = 16.42, CI 3.57–104.73, p = 0.003) as independent predictors of a fatal outcome.

**Table 6:** Prognostic indicators of death at the time of admission

Outcome	Cases of death		aOR^*^	[CI** 95%]	p-value
	n	%			
Coma	10	47.6	15.54	[5.43–44.41]	< 0.01
Hypoglycemia	4	57.1	15.37	[2.17–65.3]	< 0.01
Convulsion	8	15.1	2.11	[0.82–5.40]	0.12
Respiratory distress	10	20.8	3.85	[1.53–9.73]	0.004
Severe anemia	11	6.9	0.24	[0.08–0.68]	0.51
Prostration	11	8.5	0.76	[0.31–1.86]	0.54
Anormal bleeding	3	60	16.42	[3.57–104.73]	0.003
Hyperlactatemia	6	10.2	1.02	[NAN–INF]	0.97
Hyperparasitemia	8	22.2	0.88	[0.34–2.30]	0.37
Practice of self-medication	19	11.1	2.08	[0.42–10.36]	0.16

aOR^*^ Ajust Odd ratio, IC^**^ confidence interval

## Discussion

Malaria is one of the world’s leading parasitic diseases and affects a considerably large number of people especially children under 5 years old. This study was designed to describe the epidemiology, clinical and laboratory presentations of severe falciparum malaria according to the last WHO updates [2] in patients at the emergency ward of the CHRAB hospital, in order to improve the diagnosis, classification and appropriate management of malaria.

Malaria prevalence was 18.9%, this prevalence has remained stable in Franceville since 2011 [5, 10, 11]. Of the 1065 patients with malaria, 220 cases of severe malaria were admitted for hospitalization corresponding to 20.6% of overall malaria cases. A similar finding has been noted in Gabon (Libreville) and Cameroon [12, 13]. Other studies in West Africa [14, 15] showed lower values than those of this study (4.4% and 6.4%, respectively). This difference could be explained by the difference in epidemiological pattern. Indeed the central African countries have a hyperendemic or holendemic facies while West African countries have a seasonal pattern. Higher incidences have also been reported in other studies done in Cameroon and Central Africa Republic [16–18]. Despite the fact that these studies were carried out in central Africa, the study settings differed, as well as methodologies and sample sizes, which could explain the variations in the incidences.

In this study, the most represented age group was 0–5 years i.e. three quarters (75.0%) of population. This finding is in accordance with other studies, which states that children less than 5 years are most vulnerable to malaria [12, 19, 20, 21, 22]. Most of the participants (77.7%) used self-medication. This findings can result in delayed treatment of uncomplicated *P. falciparum* malaria with further progression to severe malaria. In this study the mean time between the onset of the disease and admission was 3.5 ± 1 days (extremes 1–7 days). Indeed, several studies in other African settings have reported an increased risk of severe malaria with delay [23–25] in presentation.

The frequency of severe falciparum malaria cases was associated with pluviometry, with most cases seen in the months of September to November (rainy season in Gabon). This result is in accordance with another study performed by Maghendji-Nzondo et al. [5]. This observation was also noted in Cameroon by Chiabi et al. [17]. This result could be explained by the fact that vector of the malaria parasite in man, is a mosquito species (*Anopheles gambiae species complex.*) which develops during the rainy season. Rains create a moist climate, resulting in a rapid parasitic cycle in the mosquito, thus increasing malaria transmission during this period.

The recent definition of severe malaria requires additional investigations that are difficult to carry out sub-Saharan countries, especially in an emergency situation. In this context, the study was unable to diagnose metabolic acidosis. This difficulty is reported by Camara et al. [15] in Senegal, by Ndoyo et al. [26] in Central Africa Republic, and by Sanou et al. [27] in Ouagadougou [27].

The most frequent clinical feature of severe malaria on admission was neurological disorders, particularly prostration and convulsion, followed by severe anaemia, hyperlactataemia and respiratory distress. Similar results were also observed by other authors [13, 19, 21, 28, 29, 30]. Hypoglycaemia, haemoglobinuria and renal failure were less common in this study. Other studies led in Senegal, Central Africa and Burkina-Faso have confirmed their rarity [15, 26, 27].

The independent prognostic factors associated with mortality in this study were coma, respiratory distress, hypoglycaemia and abnormal bleeding. These observations are entirely consistent with other studies [31]. The severity of impaired consciousness was measured using the Blantyre or Glasgow coma scale. Several studies have shown that impaired consciousness and seizures are associated with a higher risk of mortality in malaria patients, especially in children [19, 32]. The results of this study showed that respiratory distress was associated with mortality. In childhood malaria, respiratory distress is largely attributed to metabolic acidosis [33], unfortunately in the present study measure the metabolic acidosis could not be realized. A previous study revealed that the incidence of pulmonary dysfunction is 3% to 10% in patients with *P. falciparum* infections, and the mortality rate is approximately 70% [34] that confirming findings obtained in the present study. Many studies have also demonstrated hypoglycaemia as an important risk factor for death [15, 19, 35] and the present study have made the similar observation. Imbert and Camara, noted the very poor prognosis of hypoglycaemia because its clinical expression can be masked by possible neurological disorders and it aggravates the latter [15, 36]. Hypoglycaemia is often undervalued by clinicians that could explain the rarity of cases [15].

This study highlight only 5 cases of abnormal bleeding but this feature was associated with mortality. It is also rare in other studies. Severe anaemia was the second most frequent clinical feature of severe malaria in this study but was associated with decreased mortality. A similar observation in other studies showed a better outcome in children with severe anaemia [19, 30]. This result could be explain by the fact that the patients with severe anaemia were rapidly transfused and thus correcting their anaemia.

## Conclusion

At the CHRAB hospital, severe malaria is one of the main cause of hospitalization and its related mortality is high The most common clinical features of severe malaria in this study were neurological disorders, severe anaemia and respiratory distress and coma, respiratory distress, hypoglycaemia and abnormal bleeding were independent prognostic factors associated with mortality in this study.

## Data Availability

Not applicable.

## Abbreviations

ACT: Artemisinin-based combination therapy
ASECNA: Agence pour la sécurité de la navigation aérienne en Afrique et à Madagascar
CANTAM: Central Africa network on tuberculosis, HIV/AIDS and malaria
CIRMF: Centre International de Recherches Médicales de Franceville
CHRAB: Centre Hospitalier Régional Amissa Bongo
EDCTP: European and Developing Countries Clinical Trials Partnership
RDT: Rapid diagnostic test
WHO: World Health Organization
